# Evaluation of National Event-Based Surveillance, Nigeria, 2016–2018

**DOI:** 10.3201/eid2703.200141

**Published:** 2021-03

**Authors:** Kazim Beebeejaun, James Elston, Isabel Oliver, Adachioma Ihueze, Chika Ukenedo, Olusola Aruna, Favour Makava, Ejezie Obiefuna, Womi Eteng, Mercy Niyang, Ebere Okereke, Bola Gobir, Elsie Ilori, Olubunmi Ojo, Chikwe Ihekweazu

**Affiliations:** Public Health England, London, UK (K. Beebeejaun, J. Elson, I. Oliver, O. Aruna, E. Okereke);; University of Maryland Baltimore, Abuja, Nigeria (A. Ihueze, C. Ukenedo, F. Makava, M. Niyang, B. Gobir);; Nigeria Centre for Disease Control, Abuja (E. Obiefuna, W. Eteng, E. Ilori, O. Ojo, C. Ihekweazu)

**Keywords:** public health surveillance, outbreaks, internet, global health, Nigeria, viruses, bacteria, Lassa fever, cholera

## Abstract

Nigeria Centres for Disease Control and Prevention established an event-based surveillance (EBS) system in 2016 to supplement traditional surveillance structures. The EBS system is comprised of an internet-based data mining tool and a call center. To evaluate the EBS system for usefulness, simplicity, acceptability, timeliness, and data quality, we performed a descriptive analysis of signals received during September 2017–June 2018. We used questionnaires, semistructured interviews, and direct observation to collect information from EBS staff. Amongst 43,631 raw signals detected, 138 (0.3%) were escalated; 63 (46%) of those were verified as events, including 25 Lassa fever outbreaks and 13 cholera outbreaks. Interviewees provided multiple examples of earlier outbreak detections but suggested notifications and logging could be improved to ensure action. EBS proved effective in detecting outbreaks, but we noted clear opportunities for efficiency gains. We recommend improving signal logging, standardizing processes, and revising outputs to ensure appropriate public health action.

In resource-limited settings, classical indicator-based surveillance approaches can be limited by available diagnostic capacity and surveillance architecture (*1**–**3*). The Ebola outbreak in West Africa during 2014–2016 highlighted surveillance needs and generated sustained commitment to global health security with a focus on the implementation of the International Health Regulations (IHR 2005) (*4*). The World Health Organization considers implementation of event-based surveillance (EBS) a major priority for developing countries worldwide and a critical component for meeting IHR (2005) commitments (*5*,*6*).

EBS is the organized and rapid capture of information about events that are a potential risk to public health (*7*). Information captured by EBS can include rumors and other ad hoc reports from indirect channels, such as news organizations or social media, and direct channels, such as reporting by members of the public or healthcare workers. Events of interest include those related to the occurrence of disease in humans, including clustered cases of a disease or syndrome; unusual disease patterns or unexpected deaths identified by health workers and other key informants; diseases and deaths in animals; contaminated food products; and water and environmental hazards (*7*).

EBS systems have been implemented across Africa but most are at the community level (*8*–*11*). Supporting the implementation of EBS at a national level is a priority for the Africa Centres for Disease Control and Prevention (Africa CDC), which aims for >60% of member states to have an established EBS system by 2021. Africa CDC has proposed frameworks to support this implementation (*12*). Sharing knowledge and best practices from the few existing national EBS systems implemented in Africa is crucial for informing this process.

The Nigeria Centres for Disease Control and Prevention (NCDC) introduced EBS in 2016. NCDC EBS was supported by the University of Maryland Baltimore (UMB) through a grant from the US Centers for Disease Control and Prevention (CDC). The aim of the EBS is to rapidly collect and organize information about signals and trigger public health action by NCDC and its partners. Nigeria’s EBS system uses data actively mined from internet sources by Tatafo, a software platform developed by UMB for NCDC; data collected from incoming calls from the public and healthcare professionals at NCDC’s Connect Centre; and information collected by systematic and ad hoc searches of social media, blogs, health tracking websites, and the news media.

The evaluation was undertaken as part of a 4-year partnership between Public Health England (PHE), UK Department of Health (UK DoH), and NCDC to strengthen capabilities for compliance with IHR (2005). The purpose of the project was to describe the NCDC EBS system and the nature of signals and events detected; evaluate the system against its objectives and provide recommendations to improve effectiveness and efficiency and maximize utility of the system.

## Methods

### Study Design and Evaluation Period

The evaluation was performed over a 4-week period in July 2018 and informed by CDC guidelines for the evaluation of public health surveillance systems (*13*). We used a mixed methods approach comprising quantitative and qualitative data collection using semistructured interviews, document reviews, observations, questionnaires, and analysis of routinely collected data.

We conducted 19 semistructured interviews by purposive sampling of key NCDC staff members directly involved in or receiving outputs from the EBS system. Staff included call handlers, surveillance officers, data management staff, department heads, and NCDC senior leadership.

We used a bespoke topic guide to capture views on functionality, usefulness, and efficiency of the EBS. We used a questionnaire to capture specific information for certain attributes, such as ease of use, production of outputs, and acceptability of processes.

### Describing the System, Signals, and Events Detected

Existing documentation included internal guidance on implementation of EBS and technical documents on how signals were detected. Semistructured interviews explored the structure of teams, steps in escalation of signals, and data flows. Documentation was supplemented with hands-on experience working alongside and observing practices of EBS staff for 3 weeks.

### Data Sources and Links

During November 1, 2016–June 30, 2018, raw signal data were exported from the Web-based systems Tatafo and SugarCRM (https://info.sugarcrm.com). During September 1, 2017–June 30, 2018, escalated signal data were available through paper logbooks, which were digitized before analysis. We manually linked escalated signals to raw source signals. We linked escalated signals to raw source signals if the following were consistent: disease or syndrome; location or geography, such as state and town for which location information were recorded; time ±5 days; and source, such as newspaper or social media. To estimate the number of unique raw signals detected, we defined a signal cluster as linked signals on the same disease or syndrome that occurred ±2 days in the same geography (Table 1).

**Table 1:** Definition for terms used in evaluation of national event-based surveillance, Nigeria, 2016–2018*

Term	Definition
Raw signal	Communication received or retrieved from EBS system that contains data with potential to meet the WHO definition for a signal (*7*)
Signal	Raw signal reviewed by EBS technical staff who considered the signal to represent a potential acute risk to human health requiring investigation or verification according to the WHO definition†
Signal cluster	Group of signals detected by EBS system relating to same disease or syndrome and occurring within ±2 d in the same state
Escalated signal	A signal escalated and recorded by EBS technical staff to a senior surveillance officer for investigation and verification
Senior surveillance officer	Nominated member of the surveillance team responsible for investigating and verifying escalated signals
Event	A signal verified by SSO and surveillance team as an event that has potential for disease spread
*Terminology listed in order of appearance during EBS monitoring. EBS, event-based surveillance; SSO, senior surveillance officer; WHO, World Health Organization. †WHO definition states: Data and/or information considered by the Early Warning and Response system as representing a potential acute risk to human health. Signals may consist of reports of cases or deaths (individual or aggregated), potential exposure of human beings to biological, chemical, or radiological and nuclear hazards, or occurrence of natural or man-made disasters (*7*).	

## Evaluation

### Data Quality

We assessed data quality by reviewing completeness of data collected by EBS. These data included the date of raw signal detection, geolocation of the signal source, URLs of relevant websites, the related disease or nature of the event suspected, and estimated numbers of cases associated with the signal.

### Acceptability and Simplicity

We used questionnaires and semistructured staff interviews to investigate the ease of use of EBS system components, including data entry, logging of calls, prioritization of signals, escalation, and ease of producing routine outputs. We assessed acceptability by examining routine tasks performed by staff and the usefulness of routine outputs. We used Likert scales to query staff on their level of agreement to statements regarding the EBS.

### Timeliness

We assessed timeliness by measuring the number of days between individual steps in EBS processes from the initial detection of a signal indicating a potential event, to escalation, and then to investigation. We retrieved dates from relevant EBS Web-based platforms or paper logbooks, where available.

### Usefulness

We assessed usefulness by using semistructured staff interviews. We asked interviewees for their views on the usefulness of the EBS system, particularly regarding detection of events and the related public health action. We asked staff to provide examples to support their responses, where practical.

### Analysis

We manually entered questionnaire data in Excel (Microsoft Corp. https://www.microsoft.com). We used Stata version 14 (StataCorp LLC, https://www.stata.com) and Excel to clean and analyze data. We manually reviewed qualitative data from interviews and organized data into themes according to evaluation attributes by 2 investigators.

## Results

### Description of the EBS System

#### Detection of Signals

**Figure 1:**
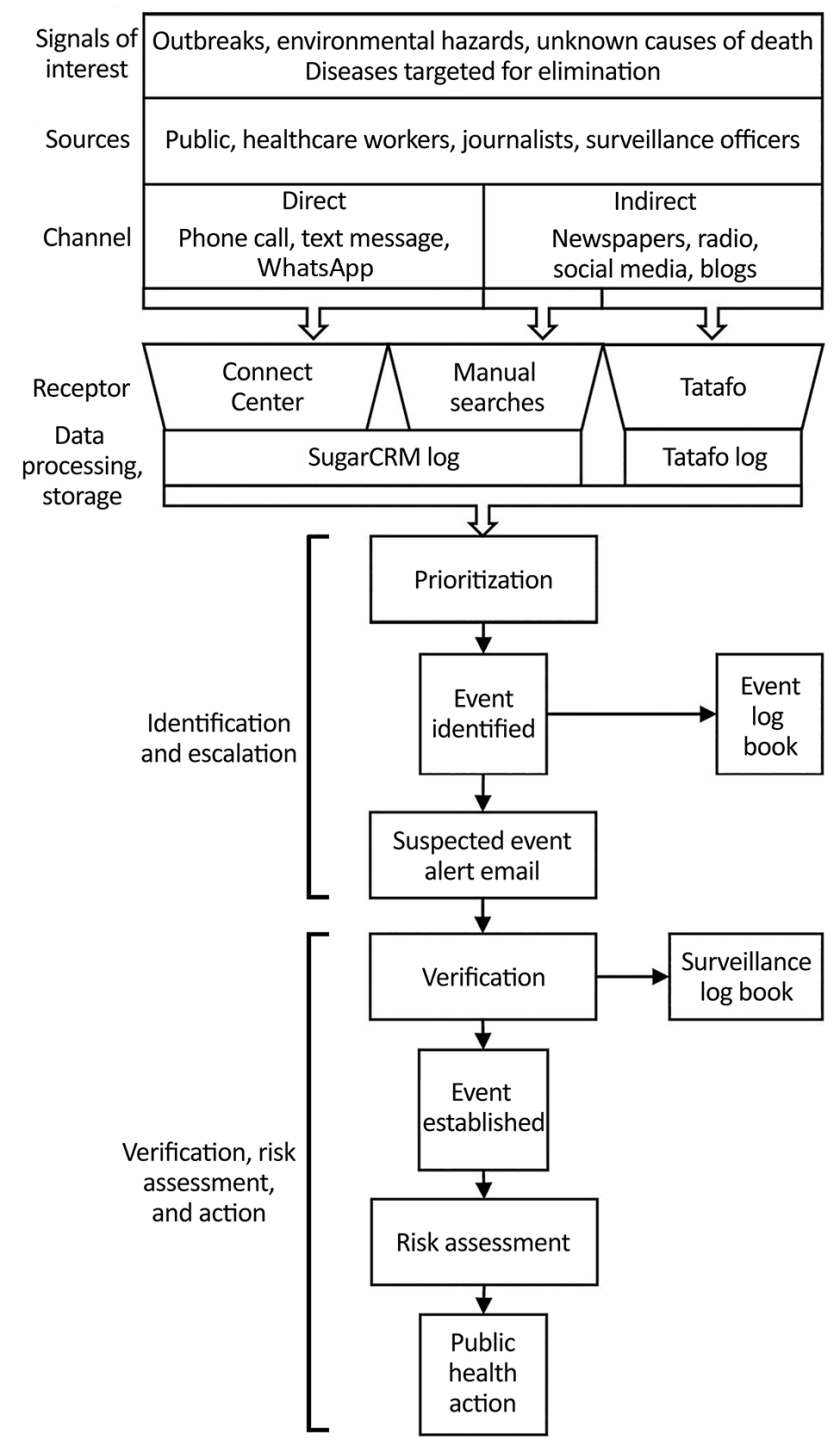
Data sources and flow of signals from detection to public health action in Nigeria Centres for Disease Control and Prevention event-based surveillance system, 2016–2018. SugarCRM, https://info.sugarcrm.com.

In accordance with the World Health Organization definition of a signal of interest (*7*), NCDC’s EBS detected signals by using 3 key receptors: Tatafo, the NCDC Connect Centre, and manual searches (Figure 1). Tatafo is an automated internet-based data system that uses text mining, text analysis, and natural language processing to detect the occurrence of events of interest from internet feeds. The system uses a list of keywords related to the 41 notifiable diseases for Nigeria (*14*). Tatafo also is customized to search for signals by using alternate terminology, such as slang and pidgin English.

The NCDC Connect Centre is the focal point of communications to and from NCDC, facilitating communications with the public, healthcare workers, and surveillance officers. The Connect Centre operated telephone, text messaging, and WhatsApp (https://www.whatsapp.com) platforms to receive signals. All communications were logged on SugarCRM.

Manual searches of online media sources included online news media websites, television, and radio. Daily online media searches were performed using a news aggregator website (http://ww38.latestnigerianews.com), which includes all major newspapers in Nigeria. Staff logged searches that had identified a signal of interest on SugarCRM.

#### Prioritization

Signals received through these channels were individually reviewed and prioritized by EBS staff according to relevance and urgency based on the potential for public health effects. Signals prioritized for escalation were forwarded to the surveillance team for further investigation and relevant public health action.

#### Escalation

Escalation was primarily performed by using a signal escalation email with details of the event sent to a predetermined distribution list that included senior surveillance officers (SSOs), technical working group (TWG) leads for the relevant disease, surveillance department leads, and the director general. SSOs acted as focal points for investigating and establishing the authenticity of an escalated signal or otherwise and performing a risk assessment. When an escalated signal was verified after initial information gathering, the verified signal was considered an event. The SSO was responsible for initiating or undertaking further investigation or public health action as appropriate for the event and recording and communicating related actions.

#### Staffing

EBS was staffed by 7 members: 2 information officers, 4 NCDC Connect Centre agents, and 1 public health analyst. These staff were funded by UMB and assigned to NCDC.

As part of their roles in EBS, 2 senior NCDC surveillance officers acted as the surveillance focal point responsible for the follow up of escalated signals. A further 12 staff were part of the surveillance department.

### Evaluation

#### Data Quality

Among raw signals detected over the 20-month evaluation period, most computer automated fields were complete, but the geolocation field was only 29% complete (20,045/69,722) (Table 2). However, a further review identified an additional 2,444 (3.5%) records that had the name of a state recorded in descriptive text fields, such as in newspaper headlines.

**Table 2:** Completeness of key fields in online event-based surveillance system, Nigeria, November 1, 2016–June 30, 2018*

EBS source	Field name	Total no. entries	No. complete entries	% Completeness
Tatafo†	Unique ID	69,722	69,722	100
	Date received	69,722	69,722	100
	Topic	69,722	69,722	100
	Headline text	69,722	69,722	100
	Website address (url)	69,722	69,722	100
	Location	69,722	20,045	29
Connect Centre	Unique ID	92	92	100
	Call category	92	92	100
	Case method	92	92	100
	Date created	92	92	100
	Date modified	92	92	100
	Description	92	92	100
	Subject	92	92	100
*EBS, event-based surveillance; ID, identification. †Tatafo is an internet data mining software platform developed by the University of Maryland Baltimore for Nigeria Centres for Disease Control and Prevention’s event-based surveillance system.				

Logs of escalated signals were maintained in Excel during September 1, 2017–December 1, 2017, and then replaced by paper logbooks. Both Excel and the paper log contained records of escalated signals detected by Tatafo or manual searches. No records of escalated signals originated from the NCDC Connect Centre, despite observations of escalations by the study team. Among the 103 escalated signals recorded by the EBS team over the evaluation period, 99 (96%) included data concerning the source of the information, 97 (94%) included the date the signal was detected, 94 (91%) contained information on action taken, 72 (70%) contained information on subsequent verification of the event, and 57 (50%) had details on the numbers of cases.

During the 20-month evaluation, SSOs kept a separate paper log containing information concerning the verification of escalated signals. Information logged included date of signal escalation, signal details, source of information, source person, investigation outcomes, and action taken. During the evaluation, SSOs logged 12 records, of which 11 (92%) contained date of signal escalation, 6 (50%) included source of information, and 5 (42%) included the name of the staff member escalating the signal. However, the original unique source identifier (ID), such as Tatafo ID or SugarCRM ID, was not logged.

#### Raw Signals Detected

During November 1, 2016–June 30, 2018, the EBS system detected 69,831 raw signals. Peaks in raw signals were observed during periods of known national disease outbreaks, including the peak of a meningitis outbreak during March–April 2017, a cholera outbreak during September 2017, a monkeypox outbreak during October 2017, and a Lassa fever outbreak during January–March 2018 (Table 3). Among raw signals, most (69,722; 99.8%) were detected by Tatafo, denoting ≈4,571 signal clusters. A mean of 3,486 raw signals (410 signal clusters) were detected by Tatafo each month. The Connect Centre received and categorized 92 communications as raw signals, of which 45% (41/92) were from phone calls and 31% (28/91) from WhatsApp messages.

**Table 3:** Number of signals detected Tatafo for top infectious disease topics, November 1, 2016–June 30, 2018*

Date raw signal detected	Top infectious disease topics											Total
	Lassa fever	HIV/ AIDS	Meningitis, CSM	Ebola	Cholera	Polio	Malaria	Monkey pox	Yellow fever	TB	Other	
2016												
Nov	6	269	4	118	33	206	166	0	3	20	496	1,321
Dec	83	727	1	51	17	63	42	0	2	3	170	1,159
2017												
Jan	206	54	3	56	1	368	39	0	6	10	437	1,180
Feb	479	440	6	42	7	79	91	0	7	7	184	1,342
Mar	749	279	832	148	102	319	83	0	12	167	200	2,891
Apr	255	385	5,116	113	44	101	372	0	9	40	288	6,723
May	374	270	1,035	1,768	76	93	210	0	11	6	178	4,021
Jun	266	469	384	106	154	167	112	0	9	37	256	1,960
Jul	215	470	89	81	215	116	199	0	14	59	389	1,847
Aug	1,308	269	48	208	116	138	241	0	10	26	262	2,626
Sep	254	266	33	152	2,100	95	166	4	209	37	470	3,786
Oct	130	300	38	268	231	399	157	3,034	357	59	1,408	6,381
Nov	36	524	18	116	102	126	331	316	42	85	716	2,412
Dec	23	1,267	43	114	210	100	186	123	400	47	485	2,998
2018												
Jan	1,494	335	49	150	92	371	115	25	615	15	379	3,640
Feb	2,008	363	67	190	41	105	186	19	152	38	436	3,605
Mar	2,812	442	91	350	139	231	212	11	104	204	742	5,338
Apr	1,216	476	48	158	244	302	1,072	33	215	45	554	4,363
May	407	585	30	2,777	676	172	456	20	40	39	705	5,907
Jun	108	489	55	264	531	195	176	35	66	63	793	2,775

*Tatafo is an internet data mining software platform developed by the University of Maryland Baltimore for Nigeria Centres for Disease Control and Prevention’s event-based surveillance system. CSM, cerebrospinal meningitis; TB, tuberculosis.

Among raw 69,831 signals, 99.8% (69,722) included pathogen information. Of raw signals with pathogen information 18% (12,429) related to Lassa fever, 12% (8,679) related to HIV/AIDS, 11% (7,990) to meningitis or cerebrospinal meningitis, 10% (7,230) to Ebola, and 7% (5,131) to cholera (Table 3).

Only 20,045 (29%) records included with geographic information, among which 22,489 referenced states (multiple states were recorded in 1,428 records). Niger State was most frequently referenced (6,032/22,489; 27%), along with Borno State (2,016/22,489; 9%), Lagos (1,928/22,489; 9%), and Federal Capital Territory (1,476/22,489; 7%). Akwa Ibom and Cross River States had no recorded signals during the study period, likely indicating a problem with search configurations in Tatafo.

#### Escalated signals

During September 1, 2017–June 30, 2018, when records were available from both EBS and SSOs, the EBS detected 43,631 raw signals, among which 138 (0.3%) were escalated to the SSOs for investigation and 75% (103/138) had details of escalation recorded. Of escalated events, 61 (44%) were from the Connect Centre, 60 (43%) from Tatafo, 2 (1%) from manual searches, and 15 (11%) had no source recorded.

**Figure 2:**
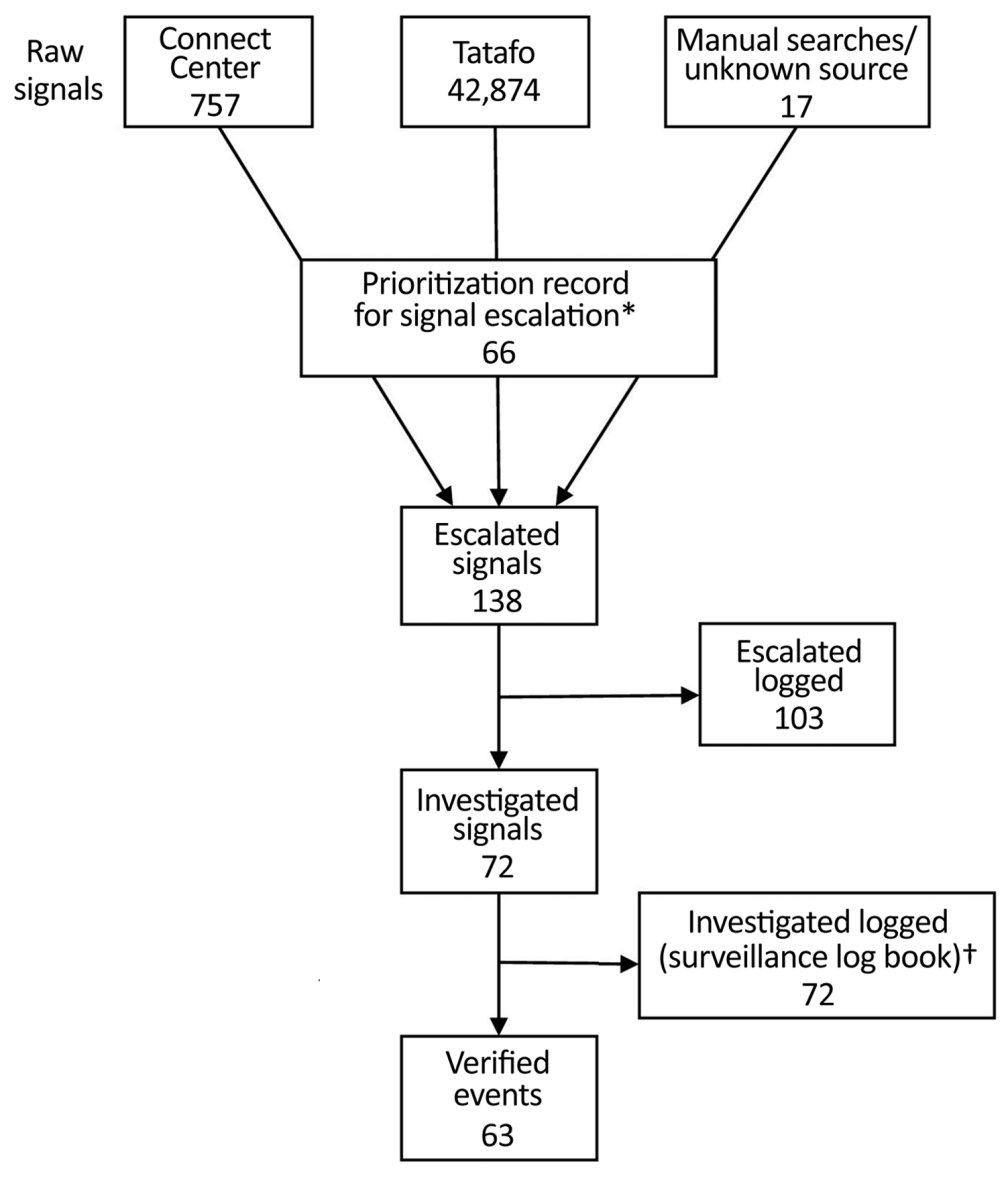
Logged recording of signals from detection to verification in event-based surveillance system, Nigeria, September 1, 2017–June 30, 2018. *Record of signal being prioritized and logged as appropriate for escalation. †For 8 additional records, it was not possible to link back to original raw signals.

Among escalated signals, EBS team logs recorded 72 (52%) for which an investigation or follow up was begun and the SSO took steps to verify the signal, but only 4 (6%) were recorded in equivalent SSO records. Among 72 recorded escalated signals, 63 (46%) were recorded as verified events in EBS team logs. The ratio of signals:verified events was 693:1 (Figure 2). Of 138 signals escalated, 66 (48%) had a record of prioritization being performed before escalation so that a record indicated that the original raw signal was triaged and logged appropriate for escalation.

#### Simplicity

In semistructured interviews, all 3 users of the Tatafo web platform agreed that the user interface was easy to navigate, data could be exported easily, and the system was reliable. However, only 2/3 users agreed that the process to prioritize raw signals for escalation was clear.

Semistructured interviews of all 4 Connect Centre staff found the system was easy or very easy to use for completing routine tasks, such as logging calls, updating records, and assigning priority levels to signals. Interviewees also indicated that it was easy to identify which senior staff members should be sent escalated signals.

#### Acceptability

Interviews with EBS staff working with Tatafo and or in the Connect Centre indicated a high level of satisfaction with the systems. Tatafo was viewed by staff to be an effective system and that it detected appropriate signals of interest. Primary EBS staff were satisfied with their roles and procedures for escalation to senior staff. However, EBS staff reported that they did not consistently receive feedback on appropriateness of escalation, progress of investigation, or outcome of escalated signals.

User satisfaction with the outputs of EBS varied according to job role. A high level of satisfaction was expressed by interviewees for the signal escalation email notifications, which were critical for action. However, several interviewees considered that notifications, although vital, were often unstructured and lacked targeting to ensure action.

Staff considered manual searches time-consuming and resource intensive. Senior staff expressed concern that the time spent on manual searches potentially wasted limited resources. EBS staff reported intermittent internet connectivity to Tatafo, and they typically lost connection once daily for <1 hour. To avoid missing signals due to intermittent connectivity, staff reported spending extra time at the office to undertake manual searches outside work hours.

#### Timeliness

Delays between detection of a raw signal and logging in Tatafo were few because the process was automated; most delays in raw signal detection could be attributed to network connectivity issues. Similarly, no delays were found between the receipt of a call by the Connect Centre and logging because phone lines were directly linked to SugarCRM. However, time of receipt of WhatsApp or text messages were not logged. Similarly, the time interval between performing a manual search and logging data could not be established due to lack of recording.

Among 79 records from which an escalated signal could be linked back to its raw signal, the median time from signal detection to escalation was 1 day (range 0–5 days) and we did not observe any date conflicts. The longest interval between signal detection and escalation observed was in February 2018 during the peak of a national Lassa fever outbreak, during which we also observed a large increase in escalated signals.

#### Usefulness

Several themes on the usefulness of the EBS emerged from interviews. Although EBS was viewed to be valuable in detecting outbreaks, users noted that a lack of recording limited oversight and assurance of action (Table 4).

**Table 4:** Assessments of national event-based surveillance system in Nigeria derived from excerpts of staff interviews*

Assessment	Staff quote (staff role)
EBS system enabled early detection of outbreaks and largely met its objectives for providing information to enable prompt identification of appropriate signals for verification and public health action	A lot of outbreaks across Nigeria are underreported. For example, if you are reported of five cases of a certain disease happening in one area, it is likely that there are actually a lot more cases in the community. The other issue is that some health facilities do not report routine data. EBS helps fill that gap. (Data manager)
Underdetection of events in areas where English was not the main spoken language	Language translation in Nigeria is an issue. There are three main languages that are competing with English. There is a large population that know how to speak and write in Hausa but cannot read or understand English. (Director)
Suboptimal recording limited effective oversight	We need something better to record what happens. When something is escalated… there needs to be an electronic record of it where I can view it and see what it is concerning and whether it has been followed up and what the action taken was. (Deputy director)

*EBS, event-based surveillance.

## Discussion

Our findings indicate that the NCDC EBS system detected events of public health concern and appropriately triggered public health investigation. Interviewees considered the EBS system useful for disease surveillance, particularly given limitations in routine integrated disease surveillance and response reporting in Nigeria. Interviewees reported that several large outbreaks were detected earlier or exclusively by EBS, primarily by Tatafo, including early detection of a large monkeypox outbreak that would not have been subject to routine surveillance. However, comparison of EBS with integrated disease surveillance and response is not practical due to lack of detailed recording of outbreaks investigated in relation to either source.

The EBS system detected signals from a range of sources, particularly from Twitter (https://www.twitter.com) and news media websites. The large number of signals verified by routine reporting and coincident surges in signals during known national outbreaks suggests the system was sensitive, however our study did not formally assess this. Of note, print newspapers, radio, and television were outside the reach of the Tatafo and the reliance of our study on internet-based media introduced some bias toward urban areas. Further, Nigeria has >520 different spoken languages; limitation to English, the official language of Nigeria spoken by ≈53% of the population, also introduced a selection bias (*15*). The fact that no signals were detected in 2 states, Akwa Ibom and Cross River, likely indicates a problem in the geographical search configurations in Tatafo. Sensitivity and timeliness of detection were therefore limited given some events would not have been detected or subject to delay until signals were in English. However, language restrictions are not unique to Nigeria’s EBS system (*16*).

Of note, no standard operating procedures (SOPs) were available, but staff appeared to have a firm understanding of data flow and communications. EBS staff had limited feedback on progress and outcomes of suspected events, verifications, and investigations, which hindered their awareness of the response. Further, prioritization of raw signals was not performed consistently, and signals often were escalated without evidence of prioritization.

During out study, only a small number of escalated signals were recorded as investigated or verified. Although our observations suggest that most escalated signals were investigated, recording was suboptimal, likely due to resource constraints and lack of SOPs. Lack of recording had implications for providing assurance of response and ensuring oversight. Suboptimal recording also limited our ability to link escalated signals to their raw signals and likely underestimated EBS related activity.

Outputs were valued by senior staff, although they considered that outputs could be better targeted to relevant persons to inform public health action. A centrally maintained directory of key staff and their disease focal points was not available to EBS staff, but a directory could have made messaging to appropriate responders more efficient.

Although interviewees indicated that several major outbreaks were detected earlier than would have been evident via routine indicator surveillance, if detected at all, we could not quantify this information by using the Salzburg standards (*17*). Time between signal detection and escalation was short, but the lack of consistent recording prevented us from estimating the time to investigation, verification, and public health intervention or action. Timeliness decreased during major outbreaks, presumably a consequence of limited resources and resource diversion from EBS to outbreak response activities. Manual searches were time consuming, resource intensive, and they yielded limited data, with only 2 signals from manual searches recorded as being escalated during the 20-month study period.

Our evaluation draws on the strengths of a mixed-methods approach to evaluate a complex surveillance system and permitted triangulation of findings. Our evaluation was subject to several limitations. The context and available data and records posed challenges in conducting a robust evaluation. For example, inclusion of a relatively small number of users introduced greater subjectivity than might have been desirable. Reporting bias is possible because staff might have avoided expressing critical opinions or might have modified aspects of their behavior in response to being observed. Although interviewees were selected purposely, a small number of senior staff were unable to be interviewed; thus, an element of selection bias could be present because of an over representation of surveillance staff. We were unable to assess the sensitivity or validity of signals because we could not establish which signals were missed by the system. Additionally, the lack of recording and volume of signals also made it difficult to determine which signals should have been investigated and required public health action. Some signals requiring investigation likely were not identified by the EBS surveillance system.

## Conclusions

Our evaluation found the NCDC EBS system to be effective in detecting relevant signals and users deemed it a valued asset for national surveillance. According to its users and NCDC leadership, the EBS system helped trigger public health action to address events of concern that otherwise might not have been detected or for which response might have been delayed. However, the extent to which investigation and response improved was difficult to establish in view of limitations in recording. EBS tasks, such as prioritization, were not performed consistently and a lack of recording hindered oversight in ensuring appropriate public health action occurred. The lack of documented SOPs potentially compromised quality and consistency of practice. Furthermore, our evaluation found that routine outputs could have been more optimally targeted to ensure action and we identified several potential inefficiencies, such as the lack of a centralized list of disease focal points.

While a valued asset, implementation and maintenance of the NCDC EBS system required funding and investments in resources, including software systems, staff, training materials. At the time of our evaluation the EBS was supported by funds from UMB and financial and personnel investments should be relevant considerations for other countries looking to adopt national EBS.

To optimize the EBS system in Nigeria, we recommended implementation of SOPs, centralized event and response logging, targeted outputs, and continuous quality improvement processes. In addition, Tatafo should be enhanced to include non-English languages. We recommend public health organizations with surveillance needs similar to those in Nigeria use our evaluation to inform implementation of national EBS systems.
